# Urine real-time polymerase chain reaction detection for children virus pneumonia with acute human cytomegalovirus infection

**DOI:** 10.1186/1471-2334-14-245

**Published:** 2014-05-08

**Authors:** Zhidai Liu, Penghui Zhang, Shi Tang, Xiaoyan He, Rong Zhang, Xinbin Wang, Zhaojian Yuan, Junjie Tan, Bin Peng, Enmei Liu, Zhou Fu, Lin Zou

**Affiliations:** 1 Center for Clinical Molecular Medicine, Children’s Hospital, Chongqing Medical University, 136 Zhongshan Er Road, Yuzhong District, Chongqing 400014, China; 2 Center for Clinical Laboratory, Children’s Hospital, Chongqing Medical University, Yuzhong District, Chongqing, China; 3 Department of Respiratory Medicine, Children’s Hospital, Chongqing Medical University, Yuzhong District, Chongqing, China; 4 Ministry of Education Key Laboratory of Development and Disorders, Children’s Hospital, Chongqing Medical University, Yuzhong District, Chongqing, China; 5 Key Laboratory of Pediatrics in Chongqing, Children’s Hospital, Chongqing Medical University, Chongqing, China; 6 Department of Health Statistics, School of Public Health, Chongqing Medical University, Yuzhong District, Chongqing, China

**Keywords:** Human cytomegalovirus, Children, Pneumonia, Urine sample, *pp65* gene

## Abstract

**Background:**

Human cytomegalovirus (HCMV) is an important pathogen of viral pneumonia in children. The diagnosis of acute HCMV infection is complicated and difficult.

**Methods:**

Clinical and laboratory data of 6063 hospitalized children with respiratory infection and 509 with respiratory virus infection alone were retrospectively analyzed. Urine and respiratory specimens of 186 hospitalized children with pneumonia were also prospectively collected. Real-time polymerase chain reaction (PCR) and a chemiluminescent assay were used to detect HCMV DNA copy number, the *pp65* gene, and HCMV IgM.

**Results:**

The patients with respiratory virus infection alone and those with pulmonary HCMV infection (n = 422) were mostly children aged <6 months old (82.91%, 422/509). The accuracy of urine HCMV DNA (82.32%) was higher than that of HCMV IgM (67.78%), indicating that PCR of urine samples is suitable for determining pediatric acute pulmonary HCMV infection. There was no significant difference in detecting HCMV DNA or the *pp65* gene between urinary and respiratory specimens (P > 0.05) in 186 pediatric pneumonia cases. The accuracy of the *pp65* gene measured in urine for determining acute pulmonary HCMV infection was the highest (93.01%).

**Conclusions:**

Our study shows a novel method for investigating acute pulmonary HCMV infection in children by using real-time PCR and non-invasive samples. This study also highlights the superiority and potential use of the *pp65* gene as an important target for the diagnosis of acute pulmonary HCMV infection.

## Background

Pneumonia is a serious and frequent cause of hospital admission in childhood. Approximately 2 million children die from pneumonia annually, and 90% of these deaths occur in developing countries [1,2]. According to the pathogen, pneumonia is divided into viral pneumonia and bacterial pneumonia. Viral pneumonia is different from that caused by bacteria, and appears as a disease associated with fever, headache, and an increased number of white blood cells (WBCs). Viral pneumonia is frequently caused by respiratory syncytial virus (RSV), influenza virus, human cytomegalovirus (HCMV), and parainfluenza virus. HCMV belongs to the Herpesviridae family, Betaherpesvirinae subfamily, and Cytomegalovirus genus, and it causes pneumonia, hepatitis, and mononucleosis [3]. HCMV pneumonia is complicated and hard to diagnose because its symptoms are similar to other viral pneumonias. HCMV leads to pneumonia when it becomes reactive and is located in the lungs. The symptoms and signs of children with HCMV pneumonia are not specific enough for diagnosis in the clinical setting.

Several laboratory tests are applied to help with diagnosis of HCMV infection, including viral isolation and culture, serology, CMV inclusion body detection, and molecular-based assays. The gold standard for diagnosis of HCMV infection is viral isolation and culture, but the requirements for this procedure are too great for popular use in clinical practice [4,5]. Viral isolation and culture are relatively intensive and time consuming. In most clinical laboratories, serological assays and polymerase chain reaction (PCR) are the main methods for detecting HCMV infection. Serological assays based on IgM and IgG detection provide a method for identifying HCMV infection, but IgM results are not reliable in certain circumstances. One of the issues is that there is no sufficient circulating antibody in child patients [6]. Moreover, the IgG serological assay is not able to show acute infection. Although the presence of HCMV inclusion bodies indicates active viral replication, the positive percentage of this method is too low to help determine HCMV infection [7]. Real-time PCR has been developed to detect HCMV because of its time-saving feature, and high sensitivity and specificity [8]. However, there are few reports on the relationship between real-time PCR results and clinical diagnosis for HCMV related to pneumonia in the pediatric population. Although the best sample for detecting HCMV pneumonia by real-time PCR is a respiratory specimen [sputum or bronchoalveolar lavage fluid (BALF)], collecting serological specimens by an invasive procedure and respiratory specimens is difficult for children, especially those younger than 1 year old. Measuring pulmonary HCMV infection by using noninvasive samples (e.g., urine) would be helpful.

A family of proteins that are localized to the HCMV tegument are called “tegument proteins” [9]. Among them, pp65 is the major tegument protein responsible for modulating the host cell immune response in infection [10]. The protein pp65 is dispensable for replication and essential for the formation of dense bodies. When HCMV invades host cells, pp65 is recognized by the host immune system to protect infected cells [11,12]. Expression of the *pp65* gene enhances HCMV reactivation. Antigenemia for pp65 antigen is widely used to detect *pp65* expression [13-15]. However, the immunofluorescent assay is objective, and its sensitivity and specificity is lower than that of real-time PCR. In clinical practice, an effective and fast assay needs to be established to identify HCMV infection in the latent or active stage.

Although the clinical and molecular characteristics of HCMV infection have been reported, few of these characteristics are known in the pediatric population with viral pneumonia alone. This study aimed to establish a new standard for detecting acute HCMV infection, because diagnosing pneumonia with HCMV infection is still difficult. In the current study, we found that real-time PCR is more specific and sensitive than a chemiluminescent assay for HCMV infection in 509 pediatric hospitalized children with pneumonia. Results from urine samples appear to be consistent with those from sputum or BALF for pneumonia with acute HCMV infection. Moreover, the *pp65* gene is a novel factor for measuring acute HCMV infection.

## Methods

### Patients

We conducted a retrospective and consecutive study in the Children’s Hospital of Chongqing Medical University, China, from April 2009 to February 2013. For the retrospective study, 6063 male and female children between 1 day and 6 years old were hospitalized for respiratory infection, and 2201 children had pneumonia. There was a total of 509 children conforming to the inclusion criteria (see “Interpretation” section below), and they were included in the retrospective study. From February 2013 to August 2013, blood, urine, and respiratory specimens (sputum or BALF) of 500 children with pneumonia were collected, and 186 of them conformed to the inclusion criteria for the prospective study (see “Interpretation” section below). The study was approved by the Ethics Committee of the Children’s Hospital of Chongqing Medical University and the reference number was chcmu-20090005. Written informed consent was obtained from the parents or legal guardians of the patients.

### Data collection

Clinical and laboratory data were collected from the medical records of the patients. The clinical data included sex, age, season, duration of illness, symptoms, signs, and X-ray image information. Laboratory data included peripheral leukocytes, neutrophil proportion, lymphocyte proportion, monocyte proportion, eosinophil proportion, copy number of HCMV DNA, and specific IgM antibody titer for HCMV.

### Interpretation

The inclusion criteria for the retrospective study were as follows. (1) Patients were included who were confirmed as having pneumonia with typical symptoms, such as cough, wheezing, sore throat and fever, as confirmed by X-ray [16]; (2) Patients were included who had a normal immune system [including WBC count, absolute lymphocyte count, and proportion of individual subpopulations of lymphocytes, such as T(CD3+), Th(CD4+), and Ts(CD8+)], as detected by flow cytometry (BD Multitest IMK Kit, USA)] [17] (data not shown); (3) Patients were included who were infected by virus alone. Sputum or blood culture was used to show no infection by bacteria, fungus, or *Mycoplasma pneumoniae* (MP) (Additional file 1: Table S1). A chemiluminescent assay (Abbott Ireland Diagnostics Division, Sligo, Ireland) and respiratory virus antigen detection kit (Diagnostic Hybrids Inc., OH, USA) were used to screen for respiratory virus infection (Additional file 1: Table S2). *Chlamydia pneumoniae* (CP) was detected by a commercial quantitative diagnostic kit with PCR fluorescence probing (Da’an Gene Co., Ltd., Guangzhou, China); (4) There was no evidence to show that the patients suffered from severe concomitant diseases and nosocomial infections; (5) The patients did not receive any antibiotics or anti-viral drugs 48 h before diagnosis; (6) Patients could be included under the condition that they only had pulmonary HCMV infection, but did not have cytomegalovirus hepatitis or encephalitis. The inclusion criteria for the prospective study were the same as those for the retrospective study.

The guidelines for diagnosis of HCMV pneumonia were based on Paya et al.’s report [18]. Briefly, the patients had typical symptoms of pneumonia. X-rays showed evidence of diffuse lesions. Sputum and blood culture were used to prove that patients were free of bacterial, fungal, and MP infections. The results of PCR, chemiluminescent assay, and detection of virus antigen were used to show that there was no CP or other viral infections. HCMV IgM or HCMV DNA was positive and HCMV IgG was negative. Moreover, antibiotic treatment was invalid, but ganciclovir treatment was valid. Acute HCMV infection in our study was defined as follows: those who were free of bacterial, MP, CP, and fungal infections (shown by sputum and blood culture, and PCR), as well as infection from other viruses (shown by chemiluminescent assay or positive respiratory virus antigen detection); positive HCMV IgM or positive HCMV DNA and negative HCMV IgG; and those receiving invalid antibiotic treatment, but valid ganciclovir treatment. Latent HCMV infection was defined as follows: those who were HCMV IgM-negative, HCMV IgG-positive, and with other viral infections (shown by chemiluminescent assay or positive respiratory virus antigen detection); those who were free of bacterial, MP, CP, and fungal infection (shown by sputum and blood culture, and PCR); and improvement without receiving ganciclovir.

According to the presence of HCMV infection, the patients were classified as the HCMV-infection group, bi/tri-virus-infection group (multiple viral infections), and the non-HCMV-infection group.

### Real-time PCR for HCMV DNA

Detection of the copy number of HCMV DNA was performed by using the HCMV DNA quantitative diagnostic kit with PCR fluorescence probing (Da’an Gene Co., Ltd.), as previously reported [19]. This technique is based on TaqMan PCR technology, and the target sequence is in a highly conserved fragment of the HCMV genome (strain AD169).

Briefly, 1 ml of urine specimen was centrifuged at 13,000 × *g*. Urine supernatant was discarded and the pellet was collected and mixed with 50 μl of DNA extraction solution. The supernatant was then collected. Respiratory specimens were liquefied with 4% NaOH, and then centrifuged at top speed. The pellet was collected and washed. The pellet was then mixed with 50 μl of DNA extraction solution, and the supernatant was collected.

A volume of 2 μl of the supernatant and 43 μl of PCR mix (Da’an Gene Co., Ltd.) were used to perform real-time PCR, using the Applied Biosystems 7500 real-time PCR system (Applied Biosystems, CA, USA) as follows: 93°C for 2 min, 10 cycles of 93°C for 45 s, and 55°C for 60s, followed by 30 cycles of 93°C for 30 s and 55°C for 45 s. For each assay, we used positive viral load standards (10^4^, 10^5^, 10^6^, and 10^7^ copies/mL), plasmid target sequence (500 copies/ml) as a positive control, ddH_2_O as a blank control, and plasmid without target sequence as a negative quality control. HCMV DNA >500 copies per ml of specimen was regarded as positive.

### Chemiluminescent microparticle immunoassay for HCMV IgM antibody

Peripheral blood specimens were used for serological tests. HCMV-specific antibody was detected using a commercial kit (Architect CMV IgM 6C16, Abbott, Sligo, Ireland) according to the manufacturer’s instructions [20]. The Architect CMV IgM assay is a two-step immunoassay for the qualitative detection and semi-quantitative determination of IgM antibodies to HCMV in human serum and plasma. The results were measured as relative light units. If the chemiluminescent signal in the specimen was equal to or greater than 1.00, anti-CMV IgM was considered positive. If the signal in the specimen was lower than 0.85, anti-CMV IgM was considered negative. If the results were uncertain, we examined a second sample within a reasonable period of time (e.g., 2 weeks) to confirm the results. In addition, RSV, adenovirus, and Epstein-Barr virus were tested by chemiluminescent assay kits from Abbott.

### Real-time PCR for the pp65 gene in clinical specimens

A total of 186 suspected HCMV-positive cases were included. Urinary and respiratory specimens of these patients were collected and DNA was extracted. The DNA purification process of urinary and respiratory specimens was the same as that mentioned above (“Real-time PCR for HCMV DNA” section). A volume of 2 μl of the resulting supernatant and 18 μl of PCR mix were used to perform real-time PCR. The PCR mixture contained 8 μl 2.5 × Real-MasterMix, 1 μl probe enhancer (Tiangen Biotech, Beijing, China), 1 μl internal TaqMan probe (20 μmol/L), 4 μl nuclease-free water, and 2 μl forward and reverse primers (10 μmol/L) each.

The sequence of the *pp65* gene was from Genebank (AY301013). The forward primer was 5′-CCC TCC GGC AAG CTC TTT-3′ and the reverse primer was 5′-CAG GTC CTC TTC CAC GTC AGA-3′. The internal TaqMan probe 5′-TGC ACG TCA CGC TGG-3′ was labeled at the 5′ end with 6-carboxy-fluorescein as the reporter and at the 3′ end with minor groove binding as the quencher [21]. The primers and probe were synthetized by Invitrogen (Life Technologies Corporation, Shanghai, China). Purified genomic DNA from the HCMV-positive specimen was the template and was amplified by PCR. The PCR product was sequenced to confirm whether the sequence was the exact target sequence, and was then ligated to the pBackZero-T vector (TaKaRa, Dalian, China); this was called the pp65-plasmid. The pp65-plasmid concentrations were measured by ultraviolet spectrophotometry to calculate the copy number of the plasmid, and to further obtain viral load standards (10^4^, 10^5^, 10^6^, and 10^7^ copies/mL) and the positive quality control (10^3^ copies/ml) for real-time PCR. Moreover, ddH_2_O as a blank control, and plasmid without a target sequence as a negative quality control, were prepared and used for each assay. PCR was performed using the 7500 real-time PCR system (Applied Biosystems, CA, USA) under the following conditions: one cycle of 50°C for 2 min and 95°C for 10 min, followed by 40 cycles of 95°C for 15 s and 55°C for 45 s. All of the standards, controls, and samples were run in triplicate.

### Statistical analysis

Quantitative data are expressed as mean ± SD if they followed a normal distribution, or are described as medians and interquartile ranges (25^th^–75^th^). Categorical data are expressed as frequencies and percentages. The relationships between acute and chronic pneumonia, pathological classification, sex, age, and HCMV infection were evaluated by logistic regression analysis. Differences in clinical symptoms of categorical variables were determined by the chi-square test. Differences in WBCs, duration of illness, and vital signs of categorical variables were determined using analysis of variance. Paired proportions of discordant serological and PCR results in the different groups were compared with the clinical diagnosis. Paired PCR results of different specimens were tested by McNemar’s test. The receiver operating characteristic curve was used to predict the *pp65* gene as a marker for identifying acute HCMV infection or latent HCMV infection in children with pneumonia. Data were analyzed by SAS 9.13 (SAS Institute, Cary, NC, USA).

## Results

### Patients and general characteristics

A total of 6063 children with respiratory infection were investigated and 509 children were included in the retrospective study according to the inclusion criteria. The general characteristics of children in our retrospective study are shown in Additional file 1: Table S3.

The age of children with respiratory infection ranged from 1 day to 6 years old. The ratio of boys to girls was 2.20:1. The median duration of illness was 7.0 days. According to Hu et al.’s report [22], the odds ratio of HCMV pneumonia is highest in children younger than 6 months old. Therefore, the children in our retrospective study were grouped into <6 months and ≥6 months. There were no significant differences in the general characteristics of children with respiratory infection, including the odds ratio of HCMV infection in different sex groups, the percentage of patients with positive clinical manifestations, the duration of different types of pneumonia, and the incidence rate of severe pneumonia among different groups (Additional file 1: Tables S4–S6).

### Classification of viral pneumonia in patients with HCMV infection

Because viral pneumonia can be classified based on the duration of illness or anatomy, we observed the differences in the duration of illness and anatomy between patients with or without HCMV infection. There were 507 cases of acute viral pneumonia or unresolved viral pneumonia, and only two cases of chronic viral pneumonia. Among the cases of acute viral pneumonia, there were 130 cases of HCMV infection and 116 cases of multiple viral infections. For 42 unresolved cases of pneumonia, there were 15 cases of HCMV infection and 17 cases of multiple viral infection (Table 1). Cases of chronic pneumonia were too few to compare, and were excluded in the next analysis. When cases of acute viral pneumonia were used as a reference, the odds ratio (OR) for the incidence of HCMV infection in unresolved pneumonia cases was 2.527 and the 95% confidence interval (CI) was 1.103–5.789 (P = 0.03). The OR for the incidence of multiple viral infection was 3.209 and the 95% CI was 1.424–7.235 (P = 0.005). These findings indicated a high percentage of HCMV infection in patients with unresolved pneumonia. For anatomical classification of pneumonia with acute HCMV infection, the percentage of interstitial pneumonia and bronchiolitis cases with acute HCMV infection was high, similar to a previous report (Table 2) [23].

**Table 1:** Acute and unresolved pediatric pneumonia and anatomy classification of different pneumonia with HCMV infection

**Diagnosis**	**Acute pneumonia (%)**	**Unresolved pneumonia (%)**	**Odds ratio**	***P *** **value**
Non-HCMV-infection (%)^a^	219 (95.6)	10 (4.4)	1.00	
HCMV-infection (%)	130 (89.7)	15 (10.3)	2.527 (1.103,5.789)	0.0284
HCMV-concurrent-infection^b^	116 (87.2)	17 (12.8)	3.209 (1.424,7.235)	0.0049
Total	465	42		

^a^Respiratory infection caused by other viruses but no bacterial infection.

^b^Respiratory infection caused by HCMV and other viruses but no bacterial infection.

**Table 2:** Anatomy classification of different pneumonia with HCMV infection

**Pneumonia subtype**	**Total**	**Non-HCMV-Infection (%)**	**HCMV-Inf-ection (%)**	**HCMV-Concu-rrent-Infection**	**Odds ratio**	***P *** **value**
Lobar pneumonia*	80	47 (58.75)	14 (17.5)	19 (23.75)	1.00	
Bronchopneu-monitis	220	106 (48.18)	81 (36.82)	33 (15.00)	1.177 (0.72,1.926)	0.5158
Interstitial pneumonia	99	37 (37.37)	31 (31.31)	31 (31.31)	2.083 (1.191,3.642)	0.0101
Bronchiolitis	110	40 (36.36)	19 (17.27)	51 (46.36)	3.045 (1.757,5.275)	<.0001

*The Lobar Pneumonia group was defined as a reference.

### Clinical characteristics and WBC counts of pneumonia patients with acute HCMV infection

Because clinical symptoms, such as a cough, sore throat, and fever, were helpful for diagnosing pneumonia, we screened these symptoms to help diagnose viral pneumonia with acute HCMV infection. The probability of occurrence of clinical symptoms, including cough, wheezing, sore throat, and dyspnea, was significantly related to HCMV infection (Table 3, P < 0.05). However, there were no significant differences in clinical symptoms, such as fever, rodding respiration, and three depressions sign of inspiration, and nasal ale flap among these three groups (P > 0.05). Moreover, most children with pneumonia appeared to have a cough, sore throat, and dyspnea. Based on our data and a previous report [24], these symptoms are not specific enough to distinguish pneumonia with acute HCMV infection from other types of pneumonia.

**Table 3:** The clinical symptoms of pneumonia patients with HCMV infection

	**HCMV-infection (%) (n = 145)**	**Non-HCMV-infection (%)**^**a **^**(n = 230)**	**HCMV- concurrent- infection**^**b **^**(n = 134)**	**Total (%) (n = 509)**	***P *** **Value**
**Clinical symptoms**					
Cough	144 (99.31)	213 (92.61)	133 (99.25)	490 (96.27)	0.0004**
Wheezing	43 (29.66)	84 (36.52)	74 (55.22)	201 (39.49)	<.0001**
Sore throat	117 (80.69)	161 (70)	115 (85.82)	393 (77.21)	0.0012**
Dyspnea	75 (51.72)	140 (60.87)	93 (69.40)	308 (60.51)	0.0104*
Rodding respiration	3 (2.07)	9 (3.91)	9 (6.72)	21 (4.13)	0.1458
Three depressions sign of inspiration	20 (13.79)	38 (16.52)	29 (21.64)	87 (17.09)	0.2097
Nasal ale flap	4 (2.76)	11 (4.78)	7 (5.22)	22 (4.32)	0.5383
Fever (>37.5°C)	21 (14.48)	15 (6.52)	11 (8.21)	47 (9.23)	0.0309
**WBC**					
WBC (×10^9^/L)	12.29 ± 5.33	11.18 ± 4.95	10.87 ± 3.85		0.0296*
Lymphocytes%	59.92 ± 18.14	58.04 ± 18.34	65.07 ± 13.93		0.0009**
Neutrophils%	34.11 ± 18.59	35.84 ± 18.8	29.50 ± 14.14		0.0042**
Monocytes%	3.61 ± 1.99	3.73 ± 2.12	3.53 ± 1.49		0.6178
Eosinophils%	2.86 ± 2.39	2.97 ± 2.44	2.71 ± 2.28		0.6037

^a^Respiratory infection caused by other viruses but no bacterial infection.

^b^Respiratory infection caused by HCMV and other viruses but no bacterial infection.

*P < 0.05, **P < 0.01.

The vital signs of the enrolled pneumonia patients did not appear to be significantly different among the three groups (P > 0.05, Additional file 1: Table S7). Therefore, diagnosis of HCMV pneumonia by only depending on the initial clinical symptoms and vital signs is insufficient.

Laboratory tests are helpful for diagnosing infectious diseases. Patients with respiratory infection are usually detected by performing a WBC count to evaluate the infection. We found that the leukocyte count and proportion of lymphocytes were higher, while the proportion of neutrophils was lower in the HCMV-infection group compared with those in the non-HCMV-infection group (P < 0.05). However, the proportion of lymphocytes and neutrophils in patients with acute HCMV infection were in the normal range (lymphocytes, 50–70%; neutrophils, 20–40%). Moreover, although minor variations were observed in the proportion of monocytes and eosinophils between children with acute HCMV infection and those with other viral infections, there was no significant difference among the groups (P > 0.05), (Table 3).

### Serological detection of IgM and detection of HCMV DNA by real-time PCR in pneumonia patients with acute HCMV infection

Recently, serological detection for IgM and real-time PCR for DNA have been widely used to help diagnosis of HCMV infection [25]. We retrospectively analyzed the results of real-time PCR and IgM assays from children with viral pneumonia. The final clinical diagnosis was set as the standard to test the accuracy of serological detection and real-time PCR [16]. The accuracy of IgM results for all of the patients was 67.78%, and it was 71.80% in the age group of <6 months and 48.28% in the age group of >6 months. The accuracy of real-time PCR for all of the patients was 82.32%, and it was 83.41% in the age group of <6 months and 77.01% in the age group of >6 months. These results showed that the accuracy of the real-time PCR assay was higher than that of the IgM assay, and the accuracy of real-time PCR was more stable in different age groups. These data suggest that real-time PCR is the preferred method to IgM serological assay for diagnosis of acute HCMV infection (Table 4).

**Table 4:** The age correlation of pneumonia patients with HCMV detected by HCMV DNA PCR and serologic tests

**Age**	**Clinical diagnosis (+)**	**Clinical diagnosis (-)**	**HCMV-IgM True positive (%)**	**HCMV-IgM True negative (%)**	**HCMV-DNA True positive (%)**	**HCMV-DNA True negative (%)**
All	279	230	190 (68.10)	155 (67.39)	238 (85.30)	181 (78.70)
<6 m	232	190	164 (70.69)	139 (73.16)	197 (84.91)	155 (81.58)
>6 m	47	40	26 (55.32)	16 (40.00)	41 (87.23)	26 (65.00)
Total	509		345 (67.78)		419 (82.32)	

### Correlation between urinary and respiratory specimens in pneumonia patients with acute HCMV infection

Urine is an easy specimen to obtain and is non-invasive for infants. For detection of HCMV infection, HCMV is known to be shed in various secretions, especially urine [26]. In the current study, urine, blood, and respiratory specimens from 30 children with pneumonia were collected. The four main genes (*pp65*, *pp71*, *pp150,* and *pp28*), which code tegument proteins and play an important role in HCMV-invading host cells, were chosen for investigation. Detection of HCMV DNA and the tegument genes by real-time PCR was performed. Only *pp65* was selected as the target because the other tegument genes were not suitable for helping with diagnosis of HCMV infection (Additional file 1: Table S8). Although the accuracy of real-time PCR in urine was high (Table 4), we further considered whether urine was able to reflect acute pulmonary infection. To test this possibility, we prospectively selected 186 suspected pneumonia patients (18 HCMV-pneumonia patients, 24 with acute HCMV infection, 123 with latent HCMV infection, and 21 with non-HCMV infection), and simultaneously collected urinary and respiratory specimens (sputum or BALF) from each patient. The individual data of the 186 suspected pneumonia patients are shown in Additional file 1: Table S9. The HCMV DNA copy numbers of these two types of samples were then detected by real-time PCR. As shown in Table 5, the copy numbers of HCMV DNA were correlated with each other. There was no significant difference in HCMV DNA copy number between urinary and respiratory specimens for each patient, suggesting that urine is a suitable sample for pediatric acute pulmonary HCMV infection.

**Table 5:** The correlation of HCMV DNA with urine and respiratory specimen

	**Urine/respiratory specimen**				***χ***^**2**^	***P *** **value**
	+/+	-/+	+/-	-/-		
HCMV-pneumonia	18 (100)	0	0	0	-	-
Virus pneumonia with acute HCMV infection	18 (75)	6 (25)	0	0	-	-
HCMV-latent-infection	39 (31.7)	3 (2.4)	6 (4.9)	75 (61.0)	1.00	0.3173
Non-HCMV-infection	6 (28.6)	3 (14.3)	0	12 (57.1)	3.00	0.0833
Total	81 (43.5)	12 (6.5)	6 (3.2)	87 (46.8)	2.00	0.1573

### Diagnosis of acute HCMV infection in clinical specimens of child pneumonia

**Figure 1:**
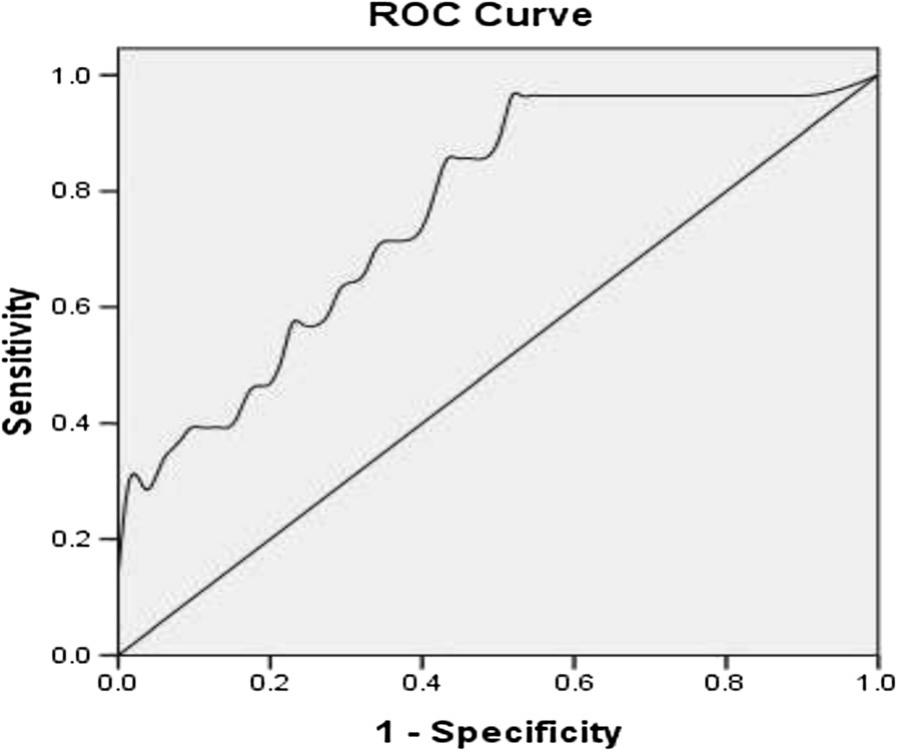
ROC analysis of pp65 gene detection between HCMV-Acute-Infection and HCMV-Latent-Infection.

In our study, the false positive and negative rates of real-time PCR for detecting the copy number of HCMV DNA were 21.0% (48/229) and 14.7% (41/279), respectively, in 509 retrospective cases (Additional file 1: Table S10). We further aimed to identify a more specific and sensitive marker for acute HCMV infection. Because *pp65* is an important gene for reflecting acute HCMV infection [10,11], we then used real-time PCR to measure the *pp65* gene in urine of children with pneumonia. There was no significant difference in *pp65* gene expression between urinary and respiratory specimens (Table 6). Interestingly, the false positive and negative rates of the *pp65* gene were only 6.9% (10/144) and 7.1% (3/42), respectively, in these patients (Table 7). This finding indicated that *pp65* was much more accurate than HCMV DNA for detecting acute HCMV infection in children with pneumonia. However, we could not identify the ideal receiver operating characteristic curve and analysis by only using the *pp65* gene from current data (Figure 1).

**Table 6:** The correlation of pp65 between urine and respiratory specimen

	**Urine/respiratory specimen (%)**				***χ***^**2**^	***P *** **value**
	**+/+**	**-/+**	**+/-**	**-/-**		
HCMV-pneumonia	18 (100)	0	0	0	-	-
Virus pneumonia with acute HCMV infection	21 (87.5)	0	0	3 (12.5)	-	-
HCMV-latent-infection	3 (2.4)	2 (1.6)	4 (3.3)	114 (92.7)	0.67	0.4142
Non-HCMV-infection	1 (4.8)	0	2 (9.5)	18 (85.7)	2.00	0.1573
Total	43 (23.1)	2 (1.1)	6 (3.2)	135 (72.6)	2.00	0.1573

**Table 7:** The correlation of pp65 with clinical diagnosis in different age

	**PP65/clinical diagnosis (%)**				***χ***^**2**^	***P *** **value**
	+/+	-/+	+/-	-/-		
<6 m	30 (22.7)	3 (2.3)	7 (5.3)	92 (69.7)	1.60	0.2059
>6 m	9 (16.7)	0	3 (5.6)	42 (77.8)	3.00	0.0833
Total	39 (21.0)	3 (1.6)	10 (5.4)	134 (72.0)	3.77	0.0522

## Discussion

For pneumonia, it is difficult to establish a “gold standard” to identify acute HCMV infection. In our study, the criteria for diagnosis of HCMV pneumonia were a positive HCMV IgM or a positive real-time PCR result for HCMV DNA, and exclusion of all bacterial, MP, and other viral infections. In our retrospective study, the incidence of HCMV pneumonia was 6.59% (145/2201) in children with pneumonia in the southwest part of China.

In our study, although the odds ratio of HCMV infection in males was slightly higher than that in females, there was no significant difference. Similar results of no significant differences in age and duration of illness were found among the different HCMV infection groups (Additional file 1: Tables S4–S5). Moreover, pediatric interstitial pneumonia and bronchiolitis were associated with a higher percentage of HCMV infection, consistent with recent reports [21].

We also demonstrated that the probability of occurrence of a cough, wheezing, sore throat, and dyspnea was different in pneumonia patients with or without acute HCMV infection. However, the differences in the rate of coughing and a sore throat among these groups were not significant enough to distinguish HCMV infection alone from multiple viral infections. Interestingly, the rate of wheezing and dyspnea was lowest in HCMV infection alone and highest with multiple viral infections. We speculate that HCMV infection does not cause wheezing and dyspnea as frequently as RSV or other viral infections. HCMV infection could alter the immune system in infants, and destroy the natural killer activity to some extent. This makes infants more vulnerable to RSV and other viruses, causing wheezing and dyspnea [27].

With regard to WBC count in our study, the proportion of lymphocytes and neutrophils in patients with acute HCMV infection was in the normal range. The results of initial clinical symptoms, vital signs, and WBCs were not specific. Therefore, new laboratory assays for determining acute HCMV infection need to be developed.

Currently, there are several types of etiological tests to detect HCMV infection. Isolation and culture of HCMV is the most specific method, but is time consuming and has low sensitivity. This method is also not suitable for routine diagnosis [4,5]. In most laboratories, serological tests are used as standard diagnostic methods. Our study showed that the accuracy of real-time PCR for HCMV DNA was higher than that of the IgM assay. The results of real-time PCR for detecting HCMV DNA were more stable in different age groups. IgM results are not reliable in children because of the immature immunological response, especially in children aged <6 months, who are the main population for acute HCMV infection [20]. Therefore, real-time PCR was superior to serology for diagnosis of HCMV infection in children younger than 6 months old.

Detection of HCMV DNA and the tegument genes (*pp65*, *pp71*, *pp150,* and *pp28*) by real-time PCR was performed. The positive rates of variables measured in peripheral blood in all of the methods were too low to help diagnose acute pulmonary HCMV infection (Additional file 1: Table S8). Moreover, peripheral blood is a difficult specimen to obtain and is invasive for children, especially for those aged <6 months. A peripheral blood specimen is not suitable for measuring infection in children. In addition, the positive rates of *pp71*, *pp150*, and *pp28* were too low to help diagnose HCMV infection when urinary and respiratory specimens were examined (Additional file 1: Table S8). Therefore, only *pp65* was selected as the target. In addition, there was no significant difference in detecting HCMV DNA and the *pp65* gene by real-time PCR between urinary and respiratory specimens. This finding indicates that urine is a suitable specimen for diagnosis of pulmonary HCMV infection, suggesting that urine could replace respiratory specimens. Taking a urine sample is non-invasive and easy to obtain, and could be used to diagnose pulmonary HCMV infection in infants with classic pneumonia symptoms.

HCMV carriers usually had positive HCMV DNA by real-time PCR in our study. However, this was result not sufficient for diagnosing acute pulmonary HCMV infection. Although the accuracy of real-time PCR for the *pp65* gene was much better than that for HCMV DNA, *pp65* gene expression alone could not be used to distinguish acute HCMV infection from latent infection. We attempted to establish a standard to distinguish acute HCMV infection from latent infection via real-time PCR for the *pp65* gene. However, we could not identify the ideal receiver operating characteristic curve and analysis by only using the *pp65* gene from current data (Figure 1). Optimized reaction methods for the *pp65* gene need to be further studied.

## Conclusions

In summary, we provide a better understanding on the incidence, and clinical and laboratory characteristics of acute HCMV infection in pediatric pneumonia. Real-time PCR is more accurate and stable than serological IgM assay for detecting acute pulmonary HCMV infection in children. More importantly, urine is a suitable sample for determining pulmonary HCMV infection in children. This indicates the potential use of this non-invasive and easy-to-obtain specimen for diagnosis of HCMV infection in pulmonary diseases. Although the *pp65* gene is a candidate marker for acute HCMV infection, the specificity and sensitivity of real-time PCR should be further optimized, and the *pp65* gene should be combined with other potential markers.

## Abbreviations

WBC: White blood cell; RSV: Respiratory syncytial virus; HCMV: Human cytomegalovirus; PCR: Polymerase chain reaction; BALF: Bronchoalveolar lavage fluid; MP: *Mycoplasma pneumonia*; CMIA: Chemiluminescent microparticle immunoassay; RLUs: Relative light units.

## Competing interests

The authors have no competing interests to declare.

## Authors’ contributions

LZ, ZF and EL participated in the conception and design of the study. ZL, PZ and BP performed the statistical analysis and interpretation of data. ZL, PZ, ST, XH, RZ, XW, ZY and JT carried out immunoassays and PCR. ZL, LZ, XH, ZF and EL drafted the manuscript. All authors read and approved the final manuscript.

## Pre-publication history

The pre-publication history for this paper can be accessed here:

http://www.biomedcentral.com/1471-2334/14/245/prepub

## Supplementary Material

Additional file 1: Table S1 The details of sputum and blood culture. **Table S2** Serologic and immunofluorescent tests for viruses detection. **Table S3**. The general and clinical characteristics of children in the retrospective study. **Table S4**. The gender and age correlation of pneumonia patients in different HCMV infection group. **Table S5**. The duration of illness in different HCMV infection group. **Table S6**. The odds ratio of severe pneumonia in different HCMV infection group. **Table S7**. The vital signs of pneumonia patients with HCMV infection. **Table S8**. PCR detection of pp65, pp71, pp150 and pp28 in periphery blood, urine and respiratory specimen from 30 children. **Table S9**. The general and clinical characteristics of children in the prospective study. **Table S10**. The correlation between results of copy number of HCMV PCR and clinical diagnosis in different age group. Click here for file

## References

[B1] DeFrances CJ Lucas CA Buie VC Golosinskiy A National Hospital Discharge Survey Natl Health Stat Report 2006 14 5 1 20 18841653

[B2] Black RE Bryce J Caulfield L Walker CF Johnson H Kalter H Katz J Liu L Walker N Bassani D Jha P Bhutta Z Eisele T Campbell H Rudan I Theodoratou E Cousens S Filippi V Ezzati M Lanata C Lawn J Peterson H Steketee R Bahl R Boerma T Cherian T Fontaine O Gore F Hutubessy R Martines J Global, regional, and national causes of child mortality in 2008: a systematic analysis Lancet 2010 14 9730 1969 1987 10.1016/S0140-6736(10)60549-1 20466419

[B3] Yurochko AD Huang ES Immunological methods for the detection of human cytomegalovirus Methods Mol Med 2000 14 1 19 21340949 10.1385/1-59259-244-9:1

[B4] Lazzarotto T Guerra B Lanari M Gabrielli L Landini MP New advances in the diagnosis of congenital cytomegalovirus infection J Clin Virol 2008 14 192 197 10.1016/j.jcv.2007.10.015 18054840

[B5] Revello MG Gerna G Diagnosis and management of human cytomegalovirus infection in the mother, fetus, and newborn infant Clin Microbiol Rev 2002 14 4 680 715 10.1128/CMR.15.4.680-715.2002 12364375 PMC126858

[B6] Emery VC CMV infected or not CMV infected: that is the question Eur J Immunol 2013 14 4 886 888 10.1002/eji.201343466 23592382

[B7] Jang HJ Kim AS Hwang JB Cytomegalovirus-associated esophageal ulcer in an immunocompetent infant: when should ganciclovir be administered? Korean J Pediatr 2012 14 12 491 493 10.3345/kjp.2012.55.12.491 23300506 PMC3534164

[B8] Ozbek SM Ozbek A Yavuz MS Detection of human cytomegalovirus and Epstein-Barr Virus in symptomatic and asymptomatic apical periodontitis lesions by real-time PCR Med Oral Patol Oral Cir Bucal 2013 14 5 e811 e816 23722135 10.4317/medoral.18905 PMC3790657

[B9] Tomtishen III Human cytomegalovirus tegument proteins (pp65, pp71, pp150, pp28) Virol J 2012 9 22 doi:10.1186/1743-422X-9-22. 22251420 10.1186/1743-422X-9-22 PMC3278345

[B10] Schmolke S Kern HF Drescher P Jahn G Plachter B The dominant phosphoprotein pp 65 (UL83) of human cytomegalovirus is dis-pensable for growth in cell culture J Virol 1995 14 10 5959 5968 7666500 10.1128/jvi.69.10.5959-5968.1995 PMC189491

[B11] Arnon TI Achdout H Levi O Markel G Saleh N Katz G Gazit R Gonen-Gross T Hanna J Nahari E Porgador A Honigman A Plachter B Mevorach D Wolf DG Mandelboim O Inhibition of the NKp30 activating receptor by pp 65 of human cytomegalovirus Nat Immunol 2005 14 5 515 523 10.1038/ni1190 15821739

[B12] Boeckh M Stevens-Ayers T Bowden RA Cytomegalovirus pp 65 Antigenemia after Autologous Marrow and Peripheral Blood Stem Cell Transplantation J Infect Dis 1996 14 5 907 912 10.1093/infdis/174.5.907 8896489

[B13] Rha B Redden D Benfield M Lakeman F Whitley RJ Shimamura M Correlation and clinical utility of pp 65 antigenemia and quantitative polymerase chain reaction assays for detection of cytomegalovirus in pediatric renal transplant patients Pediatr Transplant 2012 14 6 627 637 10.1111/j.1399-3046.2012.01741.x 22694244 PMC3461327

[B14] Goussard P Kling S Gie RP Nel ED Heyns L Rossouw GJ Janson JT CMV Pneumonia in HIV-Infected Ventilated Infants Pediat Pulmonol 2010 14 7 650 655 10.1002/ppul.21228 20575098

[B15] Manteiga R Martino R Sureda A Labeaga R Brunet S Sierra J Rabella N Cytomegalovirus pp 65 antigenemia-guided pre-emptive treatment with ganciclovir after allogeneic stem transplantation: a single-center experience Bone Marrow Transplant 1998 14 9 899 904 10.1038/sj.bmt.1701439 9827819

[B16] Lutfiyya MN Henley E Chang LF Reyburn SW Diagnosis and treatment of community-acquired pneumonia Am Fam Physician 2006 14 3 442 450 16477891

[B17] Pietras W Chaber R Pela H Trybucka K Chybicka A The recovery of immune system parameters in children following lymphoblastic leukemia therapy - preliminary report Adv Clin Exp Med 2014 14 1 97 102 24596010 10.17219/acem/37030

[B18] Ljungman P Griffiths P Paya C Definitions of cytomegalovirus infection and disease in transplant recipients Clin Infect Dis 2002 14 8 1094 1097 10.1086/339329 11914998

[B19] Zhang S Zhou YH Li L Hu Y Monitoring human cytomegalovirus infection with nested PCR: comparison of positive rates in plasma and leukocytes and with quantitative PCR Virol J 2010 14 73 10.1186/1743-422X-7-73 20398295 PMC2859376

[B20] Quinn FA Bulk Reagent Random-Access Analyzer: Architech i2000 The Immunoassay Handbook 2001 2 363 367

[B21] Paixão P Almeida S Videira PA Ligeiro D Marques T Screening of congenital cytomegalovirus infection by real-time PCR in urine pools Eur J Pediatr 2012 14 1 125 129 10.1007/s00431-011-1496-4 21614511

[B22] Vogel MN Brodoefel H Hierl T Beck R Bethge WA Claussen CD Horger MS Differences and similarities of cytomegalovirus and pneumocystis pneumonia in HIV-negative immunocompromised patients thin section CT morphology in the early phase of the disease Br J Radiol 2007 14 955 516 523 10.1259/bjr/39696316 17151065

[B23] Balasubramanian S Janakiraman L Ganesh R Deenadayalan M Naidu RK Interstitial lung disease in infancy Indian J Pediatr 2007 14 7 637 639 10.1007/s12098-007-0113-z 17699971

[B24] Xiao YM Hu ZH Differences of clinical manifestations from cytomegalovirus infection in children of various age groups Zhongguo Dang Dai Er Ke Za Zhi 2010 14 1 21 23 Article in Chinese 20113628

[B25] Buffone GJ Frost A Samo T Demmler GJ Cagle PT Lawrence EC The diagnosis of CMV pneumonitis in lung and heart/lung transplant recipients by PCR compared with traditional laboratory criteria Transplantation 1993 14 2 342 347 10.1097/00007890-199308000-00017 8395100

[B26] Gandhoke I Aggarwal R Hussain SA Pasha ST Sethi P Thakur S Lal S Khare S Congenital CMV infection; diagnosis in symptomatic infants Indian J Med Microbiol 2009 14 3 222 225 10.4103/0255-0857.53204 19584502

[B27] Schrier RD Rice GP Oldstone MB Suppression of natural killer cell activity and T cell proliferation by fresh isolates of human cytomegalovirus J Infect Dis 1986 14 6 1084 1091 10.1093/infdis/153.6.1084 2422296

