# Supplementary material for: Urine real-time polymerase chain reaction detection for children virus pneumonia with acute human cytomegalovirus infection
Source: BMC Infect Dis. 2014 May 8;14:245. doi: 10.1186/1471-2334-14-245 (PMC4024271; doi:10.1186/1471-2334-14-245)
Supplement: Additional file 1: Table S1 — The details of sputum and blood culture. Table S2 Serologic and immunofluorescent tests for viruses detection. Table S3. The general and clinical characteristics of children in the retrospective study. Table S4. The gender and age correlation of pneumonia patients in different HCMV infection group. Table S5. The duration of illness in different HCMV infection group. Table S6. The odds ratio of severe pneumonia in different HCMV infection group. Table S7. The vital signs of pneumonia patients with HCMV infection. Table S8. PCR detection of pp65, pp71, pp150 and pp28 in periphery blood, urine and respiratory specimen from 30 children. Table S9. The general and clinical characteristics of children in the prospective study. Table S10. The correlation between results of copy number of HCMV PCR and clinical diagnosis in different age group. [file 1471-2334-14-245-S1.doc]

**Table S1 The details of** sputum and blood culture

| Methods | Bacterial detection | Candida detection |
| --- | --- | --- |
| Sputum culture | *Streptococcus pneumoniae, Staphylococcus aureus, Mycobacterium tuberculosis, Corynebacterium diphtheriae, Moraxella catarrhalis, Neisseria meningitides, Haemophilus influenzae, Klebsiella pneumoniae, Escherichiacoli, Pseudomonas aeruginosa, Acinetobacter baumanii, Legionella pneumophila and mycoplasma pneumonia* | *Candida albicans, Candida famata, Candida dubliniensis, Candida kefyr,*  *Candida glabrata, Candida krusei,*  *Candida lusitaniae, Candida norvegensis, Candida parapsilosis,*  *Candida tropicalis, Candida guilliermondii*  *Cryptococcus neoformans* |
| Blood culture | *Streptococcus pneumoniae, Staphylococcus aureus, staphylococcus epidermidis, Klebsiella pneumoniae, Escherichiacoli, Haemophilus influenzae, Neisseria meningitides and Legionella pneumophila* |  |

**Table S2 Serologic and immunofluorescent tests for viruses detection**

| Type of Tests | Viruses detected |
| --- | --- |
| Chemiluminescenct kit | human cytomegalovirus (HCMV), respiratory syncytial virus(RSV), adenovirus(ADV) and epstein-barr virus(EBV) |
| Respiratory virus antigen detection kit | RSV, ADV, influenza virus A and B, parainfluenza virus I, II and III |

**Table S3 The general and clinical characteristics of children**

**in the retrospective study**

| **Clinical or biological characteristics** | N ^a^ (%) | Value ^b^ |
| --- | --- | --- |
| All children | 509 (100%) |  |
| Gender |  |  |
| Boy | 350 (68.76) |  |
| Girl | 159 (31.24) |  |
| Age (years) |  |  |
| <6m | 422 (82.91) |  |
| >6m | 87 (17.09) |  |
| Clinical symptoms |  |  |
| Cough | 490 (96.27) |  |
| Wheezing | 201 (39.49) |  |
| Sore throat | 393 (77.21) |  |
| Dyspnea | 308 (60.51) |  |
| Rodding respiration | 21 (4.13) |  |
| Three depressions sign of inspiration | 87 (17.09) |  |
| Nasal ale flap | 22 (4.32) |  |
| Fever (>37.5°C) | 47 (9.23) |  |
| Duration of illness |  |  |
| Acute pneumonia | 465 (91.36) |  |
| Unresolved pneumonia | 42 (8.25) |  |
| Chronic pneumonia | 2 (0.39) |  |
| Vital sign |  |  |
| Temperature |  | 36.85±0.34 |
| Breathing rate |  | 48.38±6.69 |
| Heart rate |  | 132.59±8.35 |
| Leucocyte count and subtype |  |  |
| WBC count (×10^9^ /L) |  | 11.63±3.60 |
| Lymphocytes (%) |  | 61.98±13.25 |
| Neutrophils (%) |  | 32.33±13.51 |
| Monocytes (%) |  | 3.56±1.36 |
| Eosinophils (%) |  | 2.78±1.63 |
| Anatomy classification |  |  |
| Lobar pneumonia | 80 (15.72) |  |
| Bronchopneumonitis | 220 (43.22) |  |
| Interstitial pneumonia | 99 (19.45) |  |
| Bronchiolitis | 110 (21.61) |  |
| Infection status |  |  |
| Non-HCMV-Infection | 230 (45.19) |  |
| HCMV-Infection | 145 (28.49) |  |
| Bi/Tri-Viruses-Infection | 134 (26.33) |  |

^a^ Children Number; ^b^ Value and Standard Deviation

**Table S4 The gender and age correlation of pneumonia patients in different HCMV infection group**

|  | **Total** | **Non-HCMV-**  **Infection (%)^a^** | **HCMV-**  **Infection (%)** | **Bi/Tri-Viruses**  **-Infection (%)^b^** | **Odds Ratio** | ***P* Value** |
| --- | --- | --- | --- | --- | --- | --- |
| **Gender** |  |  |  |  |  |  |
| Girl* | 159 | 72 (45.28) | 45 (28.30) | 42 (26.42) | 1.00 |  |
| Boy | 350 | 158 (45.14) | 100 (28.57) | 92 (26.29) | 0.999 (0.705,1.416) | 0.9950 |
| Total | 509 | 230 | 279 | |  |  |
| **Age** |  |  |  |  |  |  |
| <6m* | 422 | 190 (45.02) | 119 (28.20) | 113 (26.78) | 1.00 |  |
| >6m | 87 | 40 (45.98) | 26 (29.89) | 21 (24.14) | 1.077 (0.7,1.656) | 0.7369 |
| Total | 509 | 230 (45.19) | 145 (28.49) | 134 (26.33) |  |  |

*The Girl group was defined as a reference. *The <6m group was defined as a reference.

^a^ Respiratory infection caused by other viruses but no bacterial infection

^b^ Respiratory infection caused by HCMV and other viruses but no bacterial infection

Tested by logistic regression analysis

**Table S5** The duration of illness in different HCMV infection group

|  | **HCMV-Infection (%)** | **Non-HCMV**  **-Infection (%)** | **Bi/Tri-Viruses-**  **Infection (%)** | ***P* Value** |
| --- | --- | --- | --- | --- |
| Number | 110 | 174 | 116 |  |
| Duration of Illness (Day) | 7.35±2.97 | 7.64±2.73 | 7.58±2.52 | 0.6757 |

Tested by ANOVA

**Table S6** The odds ratio of severe pneumonia in different HCMV infection group

| **Group** | **n** | **Severe Pneumonia** | **Incidence Rate (%)** |
| --- | --- | --- | --- |
| HCMV-Infection | 145 | 17 | 11.72 |
| Non-HCMV-Infection | 230 | 28 | 12.17 |
| Bi/Tri-Viruses-Infection | 134 | 22 | 16.42 |
| Total | 509 | 67 | 13.16 |

**Table S7 The vital signs of pneumonia patients with HCMV infection**

| **Vital sign** | **HCMV-Infection** | **Non-HCMV-Infection ^a^** | **Bi/Tri-Viruses**  **-Infection ^b^** | ***P* Value** |
| --- | --- | --- | --- | --- |
| Temperature | 36.86±0.54 | 36.83±0.51 | 36.84±0.46 | 0.8551 |
| Breathing rate | 46.94±7.91 | 48.94±11.19 | 49.71±10.54 | 0.0588 |
| Heart rate | 130.93±9.69 | 132.62±13.11 | 134.25±13.17 | 0.0782 |

^a^ Respiratory infection caused by other viruses but no bacterial infection.

^b^ Respiratory infection caused by HCMV and other viruses but no bacterial infection.

Tested by ANOVA

**Table S8 The general and clinical characteristics of children**

**in the prospective study**

| **Clinical or biological characteristics** | N ^a^ (%) | Value ^b^ |
| --- | --- | --- |
| All children | 186 (100%) |  |
| Gender |  |  |
| Boy | 123 (66.13) |  |
| Girl | 63 (33.87) |  |
| Age (years) |  |  |
| <6m | 132 (70.97) |  |
| >6m | 54 (29.03) |  |
| Clinical symptoms |  |  |
| Cough | 182 (97.85) |  |
| Wheezing | 76 (40.86) |  |
| Sore throat | 130 (69.89) |  |
| Dyspnea | 104 (55.91) |  |
| Rodding respiration | 9 (4.84) |  |
| Three depressions sign of inspiration | 34 (18.28) |  |
| Nasal ale flap | 11 (5.91) |  |
| Fever (>37.5°C) | 19 (10.22) |  |
| Duration of illness |  |  |
| Acute pneumonia | 162 (87.10) |  |
| Unresolved pneumonia | 24 (12.90) |  |
| Chronic pneumonia | 0 (0) |  |
| Vital sign |  |  |
| Temperature |  | 36.85±0.39 |
| Breathing rate |  | 46.78±6.62 |
| Heart rate |  | 131.16±7.16 |
| Leucocyte count and subtype |  |  |
| WBC count (×10^9^ /L) |  | 12.63±4.16 |
| Lymphocytes (%) |  | 55.97±16.11 |
| Neutrophils (%) |  | 38.38±16.68 |
| Monocytes (%) |  | 3.54±1.57 |
| Eosinophils (%) |  | 2.69±1.51 |
| Anatomy classification |  |  |
| Lobar pneumonia | 32 (17.20) |  |
| Bronchopneumonitis | 74 (39.78) |  |
| Interstitial pneumonia | 38 (20.43) |  |
| Bronchiolitis | 42 (22.58) |  |
| Infection status |  |  |
| Non-HCMV-Infection | 144 (77.42) |  |
| HCMV-Infection | 18 (9.68) |  |
| Bi/Tri-Viruses-Infection | 24 (12.90) |  |

^a^ Children Number; ^b^ Value and Standard Deviation

**Table S9 The correlation between results of copy numbe**r of HCMV PCR and clinical diagnosis in different age group

|  | **PCR/Clinical diagnosis (%)** | | | | **χ2** | ***P* Value** |
| --- | --- | --- | --- | --- | --- | --- |
|  | +/+ | -/+ | +/- | -/- |  |  |
| <6m | 197 | 35 | 34 | 155 | 0.0145 | 0.9042 |
| >6m | 41 | 6 | 14 | 26 | 3.2000 | 0.0736 |
| Total | 238 | 41 | 48 | 181 | 0.5506 | 0.4581 |

Tested by McNemar's Test

**Table S10 PCR detection of pp65, pp71, pp150 and pp28** in periphery blood, urine and respiratory specimen from 30 children

|  |  | Pp65 | Pp71 | Pp150 | Pp28 |
| --- | --- | --- | --- | --- | --- |
| **Blood** | Positive number of  tegument genes (%) | 2 (6.7) | 3 (10) | 0 | 0 |
|  | Positive number of  HCMV DNA (%) | 10 (33.3) | | | |
| **Urine** | Positive number of  tegument genes (%) | 10 (33.3) | 5 (16.7) | 2 (6.7) | 1 (3.3) |
|  | Positive number of  HCMV DNA (%) | 13 (43.3) | | | |
| **Respiratory specimen** | Positive number of  tegument genes (%) | 12 (40.0) | 4 (13.3) | 2 (6.7) | 1 (3.3) |
|  | Positive number of  HCMV DNA (%) | 14 (46.7) | | | |
